# Application of the emergency medical services trigger tool to measure adverse events in prehospital emergency care: a time series analysis

**DOI:** 10.1186/s12873-018-0195-0

**Published:** 2018-11-26

**Authors:** Ian Howard, Bernard Pillay, Nicholas Castle, Loua Al Shaikh, Robert Owen, David Williams

**Affiliations:** 1 Department: Hamad Medical Corporation Ambulance Service, Institution: Hamad Medical Corporation, P.O. Box 3050, Doha, Qatar; 2 Department: Improvement Capability, Institution: Institute for Healthcare Improvement, Cambridge, USA

**Keywords:** Emergency medical services, Trigger tool, Patient safety, Adverse events, harm

## Abstract

**Background:**

Emergency Care has previously been identified as an area of significant concern regarding the prevalence of Adverse Events (AEs). However, the majority of this focus has been on the in-hospital setting, with little understanding of the identification and incidence of AEs in the prehospital environment.

**Method:**

The early development and testing of Emergency Medical Services (EMS) specific triggers for the identification of AEs and Harm has been previously described. To operationalise the Emergency Medical Services Trigger Tool (EMSTT), the processes developed by the Institute for Healthcare Improvement for use with the Global Trigger Tool were adapted to a prehospital emergency care setting. These were then applied using a stepwise approach to the analysis of 36 consecutive samples of patient care records over an 18-month period (*n* = 710). Inter-rater reliability was measured for each trigger item and level of Harm classification. Total *Triggers per 10,000 Patient Encounters*, *AEs per 10,000 Patient Encounters* and *Harm per 10,000 Patient Encounters* were measured. All measures were plotted on Statistical Process Control Charts.

**Results:**

There was a high level of inter-rater agreement across all items (range: 85.6–100%). The EMSTT found an average rate of 8.20 *Triggers per 10,000 Patient Encounters*, 2.48 *AEs per 10,000 Patient Encounters* and 0.34 *Harm events per 10,000 Patient Encounters*. Three triggers: *Change in Systolic Blood Pressure Greater Than 20%*; *Temp > 38 °C without subsequent reduction*; and *SpO*_*2*_ *< 94% without supplemental Oxygen or SpO*_*2*_ *< 85% without assisted ventilation* accounted for 93% (*n* = 180) of the triggers found throughout the longitudinal analysis.

**Discussion:**

With sufficient focus on implementation and data collection, as well as the inclusion of a contextually relevant system for classifying AE/Harm, the EMSTT represents a potentially successful strategy towards identifying the rate of AEs within EMS across a large patient population with limited commitment of time and resources.

**Electronic supplementary material:**

The online version of this article (10.1186/s12873-018-0195-0) contains supplementary material, which is available to authorized users.

## Background

The identification of adverse events (AE), defined by the Institute of Medicine as “an injury caused by medical management rather than the underlying condition of the patient” [1] has become an essential component of patient safety and clinical governance within healthcare [1, 2]. Emergency care has previously been identified as an area of significant concern regarding the prevalence of AEs [1]. However, the majority of this focus has been on the in-hospital setting, with little understanding of the identification and incidence of AEs in the prehospital environment. The potential for AEs to occur within emergency medical services (EMS) is significant, given that prehospital clinicians are required to make prompt decisions, with little to no supporting patient information, and implement care with limited resources, in a multitude of challenging environments.

A number of methods that have been employed to monitor for AEs in healthcare rely on either voluntary reporting systems, direct observation, complaints, mortality and morbidity review, or patient care documentation review [3–6]. While these methods may be effective for specific patients or defined high risk procedures, there is little evidence to suggest they provide a comprehensive or robust system of AE detection [3–6]. A novel approach known as the Trigger Tool (TT) methodology has been shown to be a more time-effective, cost-effective and sensitive means of identifying AEs when compared with more traditional methods [7–9].

The early development and testing of EMS specific triggers for the identification of AEs and Harm has been previously described [10]. The primary focus for these triggers was on the the low risk/high frequency case cohort of an EMS organisation. High risk/low frequency cases such as those that make up a key clinical care pathway (e.g. ST-elevation myocardial infarction); high-risk procedures (e.g., surgical airway); the administration of high-risk medication (e.g., paralytic agents); infrequent procedures (e.g., intraosseous cannulation) and high acuity inter-facility transport cases were excluded given that they are generally included as part of a 100% audit framework, as a result of their high potential for AEs and harm. The remaining low risk/high frequency cases, which generally make up the significant majority of EMS calls, are often neglected from audit, and as such, were the target population. In addition, the focus of audit in high risk cases is largely aimed at identifying individual occurrences of AEs and harm for case/risk management purposes, whereas the purpose of the EMS trigger tool is to act as a global measure of system performance and to facilitate general system improvement.

The aim of this study was to test the use of EMS triggers, using a sampling and review methodology, and to report on the incidence rates and types of AEs and harm they identify.

## Methodology

The EMS triggers were previously developed over several derivation rounds and a single large sample analysis for performance assessment/test characteristics [10]. As a retrospective sampling strategy for the targeted identification of cases at risk for potential AEs and harm, the EMS triggers demonstrated both a superior sensitivity and specificity compared to a random sampling strategy for audit case identification (See Table 1 for EMS Trigger Items).

**Table 1:** Emergency medical services trigger tool items

Clinical Triggers	
C1	SpO2 < 94% without supplemental oxygen or < 85% without assisted ventilation
C2	Change in systolic blood pressure > 20% from first measurement
C3	Pain score > 4/10 without subsequent reduction
C4	Temperature > 38 C without subsequent reduction
C5	Increase in Early Warning Score > 1 point
Medication Triggers	
M1	Administration of opioid analgesic and Naloxone in the same patient
Procedural Triggers	
P1	Inappropriate spinal immobilisation
Return-Call Triggers	
R1	Return to same patient within 24 h following refusal of transport

In order to operationalise the EMS triggers, we adapted the processes developed by the Institute for Healthcare Improvement (IHI) for use with the Global Trigger Tool (GTT) [11], to an EMS setting. The adapted EMS trigger tool (EMSTT) process was applied using a stepwise approach, outlined by Adler et al. in their implementation of the GTT [12], to the analysis of 36 consecutive samples of patient care records over an 18-month period (*n* = 710).

### Setting

The study was conducted within the Hamad Medical Corporation Ambulance Service (HMCAS), the government-funded national ambulance service of Qatar. HMCAS is a two-tiered provider (Ambulance Paramedic and Critical Care Paramedic) that serves a population of approximately 2.6 million people with an average daily call rate of between 600 and 700 emergency and non-emergency calls. At the time of writing, HMCAS was transitioning from a paper-based care record system to an electronic patient care record.

### Institutional review board

Ethical approval to conduct the study was granted by the Medical Research Centre of the Hamad Medical Corporation, Qatar.

### Trigger tool methodology

#### Sampling

Consistent with the sampling strategy of the GTT, the EMSTT process utilises a sampling methodology of small consecutive samples over time. Cases considered for analysis included those where there was patient contact with EMS and for whom a Patient Care Record (PCR) was generated. Excluded PCRs included: those reviewed as part of a key clinical care pathway (e.g., ST-Elevation Myocardial Infarction); high-risk procedures (e.g., surgical airway); the administration of high-risk medication (e.g., paralytic agents); infrequent procedures (e.g., intraosseous cannulation); and inter-facility transport records. Of the remaining cases, two samples per month were selected by random number table for the 18-month test period (one sample for each half of the month). Each sample consisted of 30 PCRs, with the intent to review the first 20 cases, and the additional 10 PCRs included in case any of the first 20 met exclusion criteria (See Additional files 1 and 2). The sampling process was provided to a documentation clerk, who identified the PCRs meeting inclusion criteria, and supplied paper copies to the primary reviewers for review.

#### Review process

Each review round was conducted independently by two primary reviewers (IH, BP) during scheduled and protected review time. The primary reviewers were each operational Critical Care Paramedics employed by HMCAS, with > 15 years EMS experience each, who were additionally involved in clinical governance and education within the service. Individual record reviews were limited to 10 min each. Following each review round, the two primary reviewers met to compare findings, reach consensus, and summarize the results. In cases where consensus could not be reached, a third reviewer, the HMCAS Medical Director or Head of Professions (LAS/NC) were consulted to determine an outcome. Each review round was part of an iterative process and allowed for continued refinement of the EMSTT methodology and data collection processes (See Additional files 1 and 2).

#### Data collection process

Each record was manually reviewed for the presence of triggers only. If a trigger was found, the record was further reviewed for the occurrence of AEs and/or harm. Records that did not contain a trigger were not reviewed further. The National Coordinating Council for Medication Error Reporting and Prevention (NCC MERP) classification system was used to categorise AEs and harm for EMSTT positive cases. Given the in-hospital focus of the MERP system, Category C was modified to include cases where it was unclear if harm had occurred given the short duration of care provided by EMS (Table 2) [13]. Beginning with Sample 10 (Month 5) an additional, EMS specific classification system developed by Patterson et al. [14], the Adverse Event Severity Rating Index, was introduced to run concurrently with the NCC MERP system (Table 2). This allowed a fit for purpose EMS-specific system to be used for AE classification, as opposed to relying solely on the in-hospital focused NCC MERP.

**Table 2:** Harm classification systems

Harm Classification System 1 – Modified NCC MERP (13)	
Category A	Circumstances or events that have the capacity to cause Error
Category B	An Error that did not reach the patient
Category C/EMS	An Error that reached the patient but did not cause Harm *(EMS - the potential for harm to occur was present, but could not be conclusively determined based on the short duration of exposure to EMS)*
Category D	An Error that reached the patient and required monitoring or intervention to confirm that it resulted in no Harm to the patient
Category E	Temporary Harm to the patient and required intervention
Category F	Temporary Harm to the patient and required initial or prolonged Hospitalization
Category G	Permanent patient Harm
Category H	Intervention required to sustain life
Category I	Patient death
Harm Classification System 2 – AE Severity Rating Index (14)	
Category 1	AE with Harm as a result of commission
Category 2	AE with Harm as a result of omission
Category 3	AE with Harm, but no fault
Category 4	AE with potential to cause Harm as a result of commission
Category 5	AE with potential to cause Harm as a result of omission
Category 6	AE with potential to cause Harm with no fault
Category 7	No AE identified

### Consecutive sample analysis

The EMSTT was applied over an 18-month period as part of a consecutive sample analysis using the operational processes reported above. Records for review were identified via the HMCAS centralised electronic database (Microsoft Access 2010, Redwood, WA) and cases meeting exclusion criteria were removed prior to sampling. All data was captured on a standardised data capture template and summary report template (Microsoft Excel 2010, Redwood, WA) for analysis and reporting.

#### Data analysis and reporting process

Inter-rater reliability was measured for each trigger item and level of classification overall. In addition, univariate descriptive analysis was conducted for all continuous and categorical variables (i.e.: trigger items and levels of harm classification). Lastly, a reporting standard was developed that included outcome measures for future potential benchmarking purposes and data visualisation methods for analysis and performance monitoring. The three outcome measures identified included: *Triggers per 10,000 Patient Encounters*, *AEs per 10,000 Patient Encounters* and *Harm per 10,000 Patient Encounters*. For the purpose of the reporting standard, a patient encounter was defined as: “*An interaction between a patient and EMS healthcare provider(s) for the purpose of providing healthcare service(s) or assessing the health status of a patient.*” All outcome measures were calculated and plotted on Statistical Process Control (SPC) Charts using Minitab Version 17 (2010, State College PA) and employed the Nelson Rules for detecting special cause variation [15] SPC is a branch of statistics that combines time series analysis methods with graphical presentation of data to understand variation and yield insight into whether the variation is due to chance or assignable causes [16]. The primary tool of SPC, the control chart, provides researchers and practitioners with a method of better understanding and communicating data from healthcare improvement efforts by enabling the study of variation and differentiation of special from common cause variation [17]. The data presented in this study meets all requirements associated with the use of U-charts [15].

## Results

The EMSTT identified 194 individual triggers among the 720 sampled PCRs. There was a high level of inter-rater agreement across all items ranging from 85.6–100% depending on the trigger item (Table 3). Out of a total of 40 items analysed, 4 were observed to have poor reliability (*κ* < 0.41), 8 were observed to have moderate reliability (*κ* = 0.41–0.6), 3 substantial reliability (*κ* = 0.61–0.8), and 14 near perfect reliability (*κ* = 0.81–1) [18]. Six cases in total required referral to a third reviewer, all of which were from the *Clinical* group. All disagreements were primarily attributed to PCR illegibility.

**Table 3:** Inter-rater reliability

	Percent Agreement	Cohen’s Kappa	N Agreements	N Disagreements	N Cases	N Decisions
C1	97.50%	0.297	692	18	710	1420
C1 Adverse Event	97.20%	0.276	690	20	710	1420
C1 Harm	97.20%	0.273	690	20	710	1420
C1 Harm Category 1	96.80%	0.169	687	23	710	1420
C1 Harm Category 2	98.20%	1	551	10	561	1122
C2	88.30%	0.52	627	83	710	1420
C2 Adverse Event	86.50%	0.456	614	96	710	1420
C2 Harm	87.70%	0.501	623	87	710	1420
C2 Harm Category 1	85.60%	0.431	608	102	710	1420
C2 Harm Category 2	97.10%	1	545	16	561	1122
C3	99.40%	1	706	4	710	1420
C3 Adverse Event	99.40%	1	706	4	710	1420
C3 Harm	99.40%	1	706	4	710	1420
C3 Harm Category 1	99.40%	1	706	4	710	1420
C3 Harm Category 2	100%	undefined*	561	0	561	1122
C4	100%	1	710	0	710	1420
C4 Adverse Event	99.70%	0.666	708	2	710	1420
C4 Harm	99.60%	0.499	707	3	710	1420
C4 Harm Category 1	99.60%	0.499	707	3	710	1420
C4 Harm Category 2	99.50%	1	558	3	561	1122
C5	96.90%	0.759	688	22	710	1420
C5 Adverse Event	94.40%	0.571	670	40	710	1420
C5 Harm	96.30%	0.717	684	26	710	1420
C5 Harm Category 1	93.40%	0.488	663	47	710	1420
C5 Harm Category 2	95.50%	1	536	25	561	1122
M1	100%	undefined*	710	0	710	1420
M1 Adverse Event	100%	undefined*	710	0	710	1420
M1 Harm	100%	undefined*	710	0	710	1420
M1 Harm Category 1	100%	undefined*	710	0	710	1420
M1 Harm Category 2	100%	undefined*	561	0	561	1122
P1	99.70%	1	708	2	710	1420
P1 Adverse Event	99.70%	1	708	2	710	1420
P1 Harm	99.70%	1	708	2	710	1420
P1 Harm Category 1	99.70%	1	708	2	710	1420
P1 Harm Category 2	99.30%	1	557	4	561	1122
R1	100%	undefined*	710	0	710	1420
R1 Adverse Event	100%	undefined*	710	0	710	1420
R1 Harm	100%	undefined*	710	0	710	1420
R1 Harm Category 1	100%	undefined*	710	0	710	1420
R1 Harm Category 2	100%	undefined*	561	0	561	1122

Consistent with the results reported in the development of the EMSTT, the *Clinical* group made up the majority of triggers found in our analysis [*n* = 190 (97.9%)] (Table 4).Three triggers: *Change in Systolic Blood Pressure Greater Than 20%*; *Temp > 38 °C without subsequent reduction*; and *SpO*_*2*_ *< 94% without supplemental Oxygen or SpO*_*2*_ *< 85% without assisted ventilation* accounted for 92.8% (*n* = 180) of the triggers found throughout the analysis. *Change in Systolic Blood Pressure Greater Than 20%* was the most common trigger amongst the *Clinical* group, as well as overall [*n* = 108 (55.7%)].

**Table 4:** Results summary 1 – trigger items

Trigger Items			
Trigger Item	Trigger (%)	Adverse Event (%)	Harm %
C1 - SpO2 < 94% without supplemental oxygen or < 85% without assisted ventilation	10 (5.2)	7 (3.6)	1 (0.5)
C2 - Change in systolic blood pressure > 20% from first measurement	108 (55.7)	14 (7.2)	4 (2.1)
C3 - Pain score > 4/10 without subsequent reduction	3 (1.5)	1 (0.5)	0 (0.0)
C4 - Temperature > 38 C without subsequent reduction	7 (3.6)	4 (2.1)	2 (1.0)
C5 - Increase in Early Warning Score > 1 point	62 (32.0)	27 (13.9)	1 (0.5)
M1 - Administration of opioid analgesic and Naloxone in the same patient	0 (0.0)	0 (0.0)	0 (0.0)
P1 - Inappropriate spinal immobilisation	4 (2.1)	4 (2.1)	0 (0.0)
R1 - Return to same patient within 24 h following refusal of transport	0 (0.0)	0 (0.0)	0 (0.0)

**Fig. 1:**
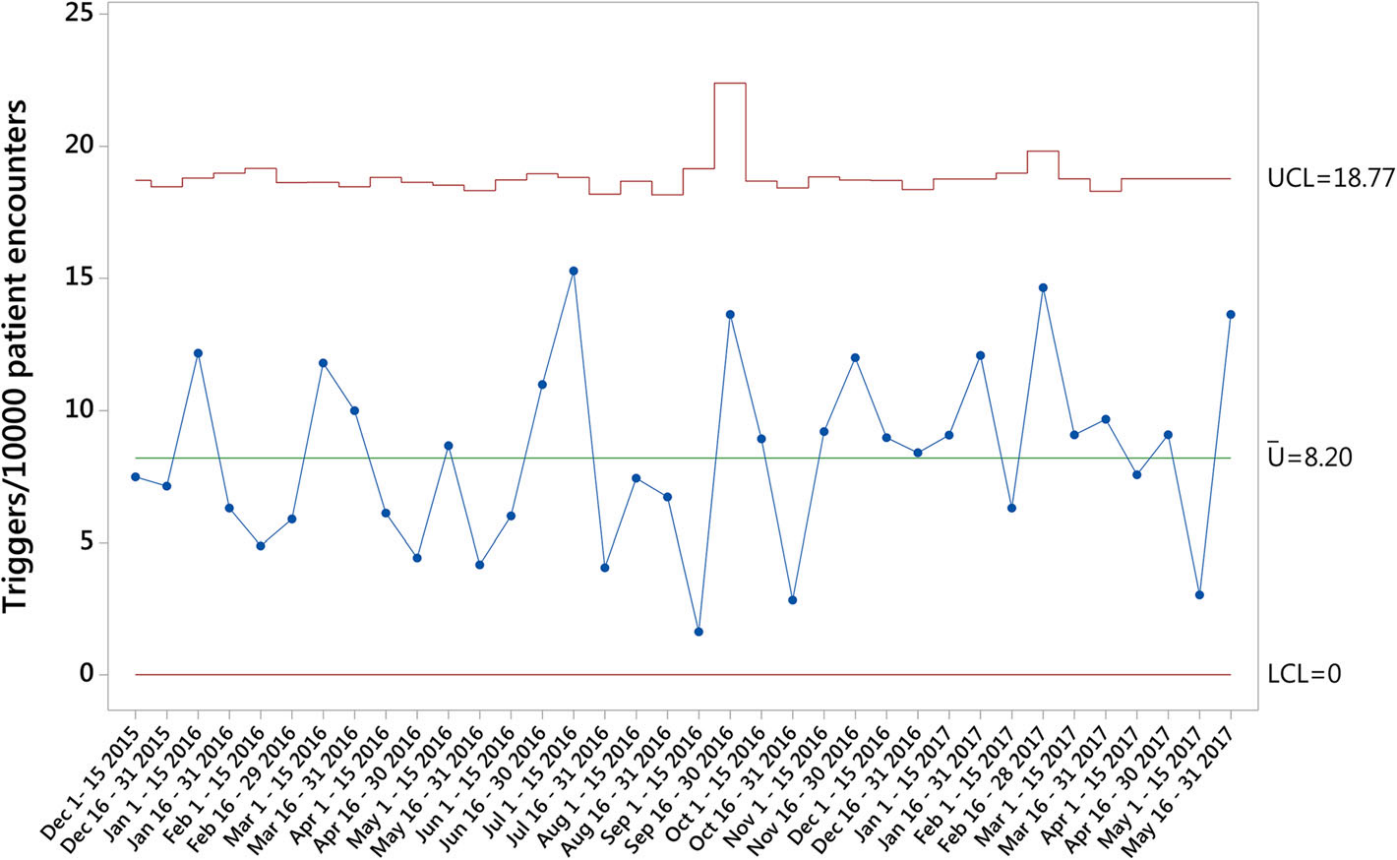
Triggers per 10,000 Patient Encounters

**Fig. 2:**
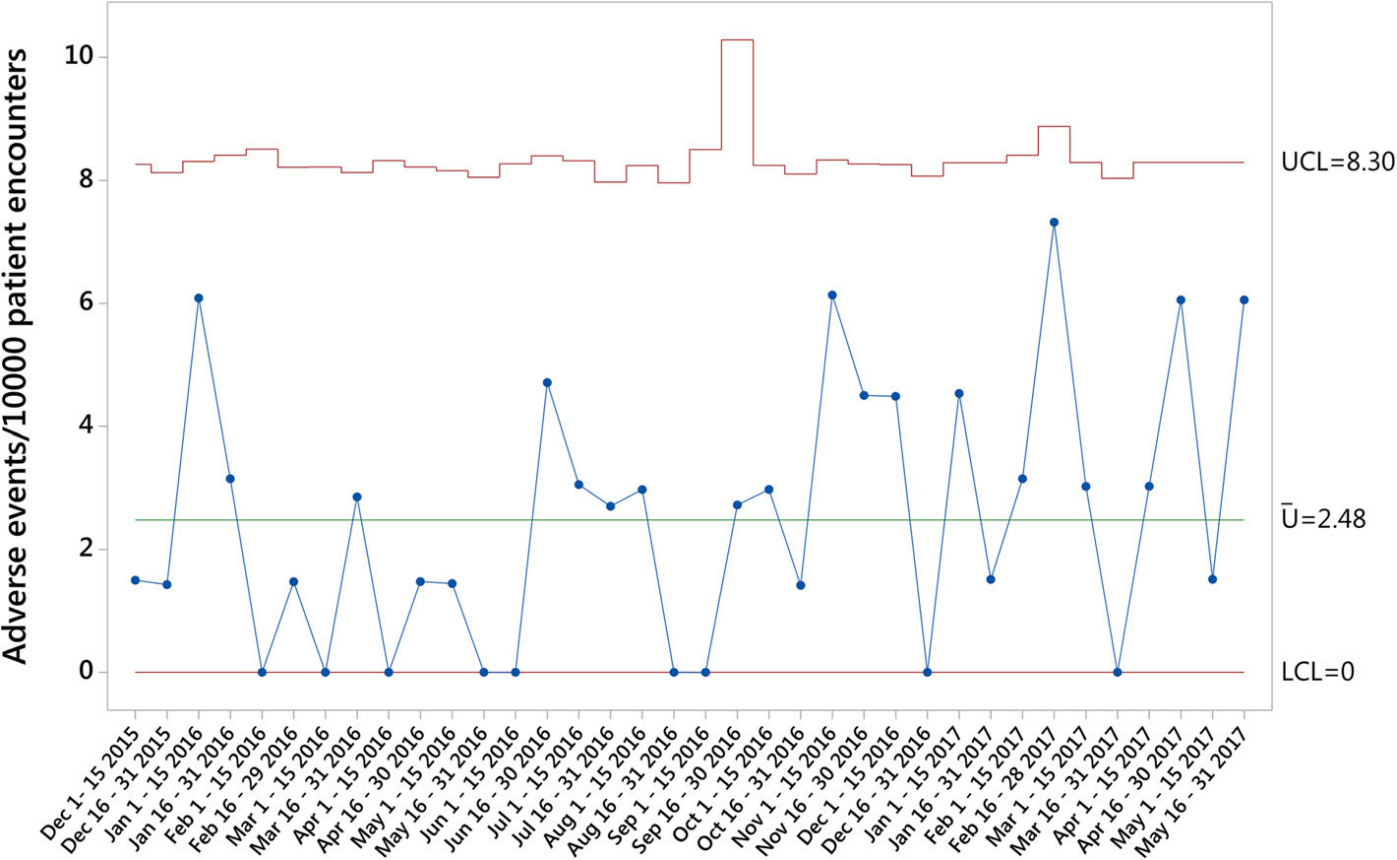
Adverse Events per 10,000 Patient Encounters

**Fig. 3:**
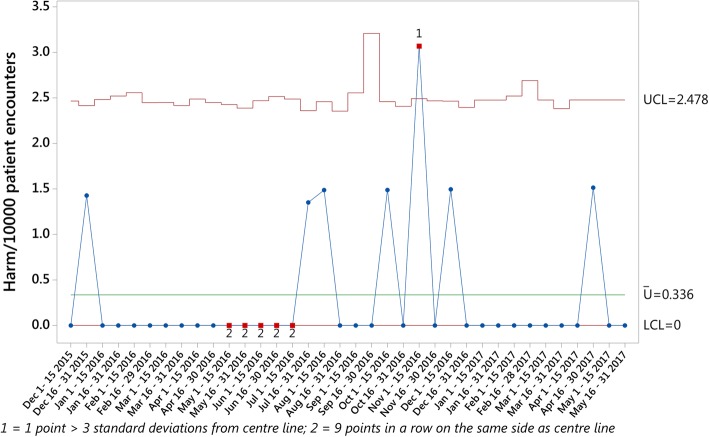
Harm per 10,000 Patient Encounters

The three primary outcomes measures were calculated and plotted on U charts for the study period. An average rate of 8.20 *Triggers per 10,000 Patient Encounters* (Fig. 1), 2.48 *AEs per 10,000 Patient Encounters* (Fig. 2) and 0.34 *Harm events per 10,000 Patient Encounters* (Fig. 3) were found during the analysis. While one sample generated data outside the control limits in the *Harm events per 10,000 Patient Encounters* chart, no obvious attributable cause for this variation could be immediately identified.

When classifying harm using the NCC MERP Category A (*Circumstances or Events that have the capacity to cause error*) was the most common assigned class [*n* = 147 (20.7%)] (Table 5). In total, only three NCC MERP categories were assigned to all cases in which triggers were found: Category A, Category B (*An error occurred but the error did not reach the patient*) [*n* = 36 (5.1%)]; and Category C (*An error occurred that reached the patient, but did not cause patient harm*) [*n* = 11 (1.5%)], modified in this study to include cases in which it was unclear if harm had occurred due to the short duration of care provided by EMS. As with the development of the EMSTT, for reporting and analysis purposes, the latter classification was counted as positive for the occurrence of Harm.

**Table 5:** Results summary 2 – harm classification system 1

Harm Classification System 1 – NCC MERP											
Classification 1	No trigger (%)	A (%)	B (%)	C/EMS (%)	D (%)	E (%)	F (%)	G (%)	H (%)	I (%)	Total (%)
C1 - SpO2 < 94% without supplemental oxygen or < 85% without assisted ventilation	700 (98.6)	6 (0.8)	3 (0.4)	1 (0.1)	0 (0.0)	0 (0.0)	0 (0.0)	0 (0.0)	0 (0.0)	0 (0.0)	10 (1.4)
C2 - Change in systolic blood pressure > 20% from first measurement	602 (84.8)	94 (13.2)	10 (1.4)	4 (0.6)	0 (0.0)	0 (0.0)	0 (0.0)	0 (0.0)	0 (0.0)	0 (0.0)	108 (15.2)
C3 - Pain score > 4/10 without subsequent reduction	707 (99.6)	2 (0.3)	1 (0.1)	0 (0.0)	0 (0.0)	0 (0.0)	0 (0.0)	0 (0.0)	0 (0.0)	0 (0.0)	3 (0.4)
C4 - Temperature > 38 C without subsequent reduction	703 (99.0)	2 (0.3)	3 (0.4)	2 (0.3)	0 (0.0)	0 (0.0)	0 (0.0)	0 (0.0)	0 (0.0)	0 (0.0)	7 (1.0)
C5 - Increase in Early Warning Score > 1 point	648 (91.3)	41 (5.8)	17 (2.4)	4 (0.6)	0 (0.0)	0 (0.0)	0 (0.0)	0 (0.0)	0 (0.0)	0 (0.0)	62 (8.7)
M1 - Administration of opioid analgesic and Naloxone in the same patient	710 (100.0)	0 (0.0)	0 (0.0)	0 (0.0)	0 (0.0)	0 (0.0)	0 (0.0)	0 (0.0)	0 (0.0)	0 (0.0)	0 (0.0)
P1 - Inappropriate spinal immobilisation	706 (99.4)	2 (0.3)	2 (0.3)	0 (0.0)	0 (0.0)	0 (0.0)	0 (0.0)	0 (0.0)	0 (0.0)	0 (0.0)	4 (0.6)
R1 - Return to same patient within 24 h following refusal of transport	710 (100.0)	0 (0.0)	0 (0.0)	0 (0.0)	0 (0.0)	0 (0.0)	0 (0.0)	0 (0.0)	0 (0.0)	0 (0.0)	0 (0.0)
Total		147 (20.7)	36 (5.1)	11 (10.5	0 (0.0)	0 (0.0)	0 (0.0)	0 (0.0)	0 (0.0)	0 (0.0)	194 (27.3)

When classifying harm using the AE Severity Rating Index, implemented with the fifth sample, Rating Code 7 (*No AE identified*) was the most common reported class in trigger positive cases [*n* = 67 (12%)] (Table 6). Similar to the NCC MERP system, three categories made up the majority of assigned categories in trigger positive cases, including: Code 7; Code 6 (*AE with potential to cause harm with no fault*) [n = 11 (2%)]; and Code 4 (*AE with potential to cause harm as a result of commission*) [n = 11 (2%)].

**Table 6:** Results summary 2 – harm classification system 2

Harm Classification System 2 – AE Severity Index									
Classification 2	No trigger (%)	7 (%)	6 (%)	5 (%)	4 (%)	3 (%)	2 (%)	1 (%)	Total %
C1 - SpO2 < 94% without supplemental oxygen or < 85% without assisted ventilation	556 (99.3)	3 (0.5)	0 (0.0)	0 (0.0)	0 (0.0)	0 (0.0)	1 (0.2)	0 (0.0)	4 (0.7)
C2 - Change in systolic blood pressure > 20% from first measurement	521 (93.0)	36 (6.4)	1 (0.2)	2 (0.4)	0 (0.0)	0 (0.0)	0 (0.0)	0 (0.0)	39 (7.0)
C3 - Pain score > 4/10 without subsequent reduction	557 (99.5)	3 (0.5)	0 (0.0)	0 (0.0)	0 (0.0)	0 (0.0)	0 (0.0)	0 (0.0)	3 (0.5)
C4 - Temperature > 38 C without subsequent reduction	554 (98.9)	2 (0.4)	0 (0.0)	3 (0.5)	0 (0.0)	0 (0.0)	1 (0.2)	0 (0.0)	6 (1.1)
C5 - Increase in Early Warning Score > 1 point	514 (91.8)	21 (3.8)	8 (1.4)	5 (0.9)	11 (2.0)	0 (0.0)	1 (0.2)	0 (0.0)	46 (8.2)
M1 - Administration of opioid analgesic and Naloxone in the same patient	560 (100.0)	0 (0.0)	0 (0.0)	0 (0.0)	0 (0.0)	0 (0.0)	0 (0.0)	0 (0.0)	0 (0.0)
P1 - Inappropriate spinal immobilisation	556 (99.3)	2 (0.4)	2 (0.4)	0 (0.0)	0 (0.0)	0 (0.0)	0 (0.0)	0 (0.0)	4 (0.7)
R1 - Return to same patient within 24 h following refusal of transport	560 (100.0)	0 (0.0)	0 (0.0)	0 (0.0)	0 (0.0)	0 (0.0)	0 (0.0)	0 (0.0)	0 (0.0)
Total		67 (12.0)	11 (2.0)	10 (1.8)	11 (2.0)	0 (0.0)	3 (0.5)	0 (0.0)	102 (18.2)

## Limitations

Our study separated the definitions of AEs and harm into separate concepts, aligning the definition of AE more synonymously with error. However, there is a recent trend towards the definition of AE to be used interchangeably and associated more with the definition of harm. The reasons for this are two-fold. Firstly, error reporting has become less prominent, given its perceived “dilution” of the reporting on harm events. In addition, early results using the trigger tool methodology reported a relatively low conversion of error into harm events, reducing the emphasis on reporting the incidence of error, and focusing more on identifying actual cases involving patient harm [19]. Finally, harm can occur in the absence of an error in the delivery of a patient’s care. These events began to be referred to as AEs and were seen as synonymous with harm given their untoward outcome, despite the potential absence of a trigger or error in patient care [19].

**Fig. 4:**
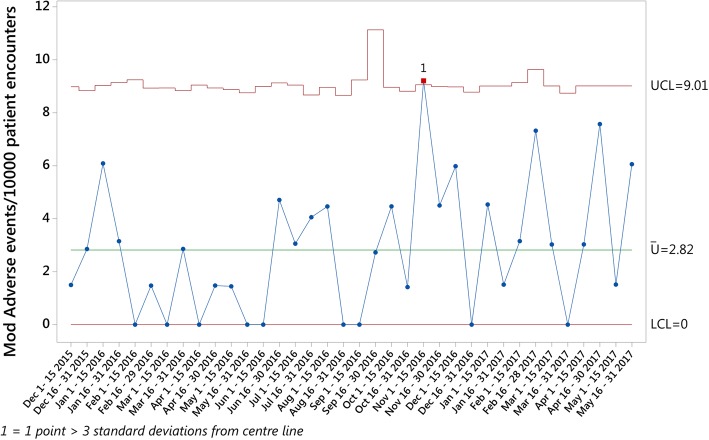
Modified Adverse Events per 10,000 Patient Encounters

In order to account for this recent change in the understanding of AEs, an additional measure was calculated to align with the evolving change in thinking towards these definitions - *Modified AEs per 10,000 Patient Encounters* values were calculated by adding AEs and harm, and the results plotted on a U chart for comparison. While this has the potential to skew the results observed for this study, the value of each of these modified measures remained relatively low, with 2.82 *Modified Adverse Events per 10,000 Patient Encounters* (Fig. 4) observed over the study period.

Consistent with the development of the EMS triggers, our study excluded several records during the sampling process, including evidence-based care pathways and high-risk/infrequent medications and procedures. Although these cases represent a small proportion of all EMS calls, the potential exists that their exclusion skewed the results of the analysis. The removal of these records was deliberate, given the aim of the tool for use in the high frequency/low risk cases often excluded from regular EMS audit programs. This limitation represents an avenue for further research and evaluation of the EMSTT in a sample where such exclusion criteria have not been applied.

Lastly, the aim of this research was limited to the development of implementation, data collection and reporting processes, and to test the EMSTT using these processes over time. As such, the study did not include tests for association or correlation between trigger items and rates of AEs and/or Harm. Similarly, this study did not include efforts aimed at improving or reducing the impact of the most common triggers. These remain as areas for future research.

## Discussion

The TT methodology has seen considerable success as a quick, reliable and simple, yet comprehensive method of detecting AEs within healthcare. These desirable characteristics have led to the successful development of trigger tools within a variety of disciplines, including general healthcare [7], surgical care [20], primary care [21], intensive care [22], paediatric care [23] as well as pharmacy [24] and laboratory services [25]. However, integrating such a system into regular operation can be a complex task and should be viewed as equally important as the content of the tool itself [11, 12]. Despite the robust planning and processes put in place, several issues became apparent that hindered data collection for several months, requiring constant intervention and refinement to correct, and included:

- Communication of the process and operational definitions emerged as a central component both internally within the project team, and externally with all departments the project could potentially impact.
- Dedicated, protected time for each step within the process was essential to complete the required reviews. When the time required to complete each review round was specifically scheduled, review rounds were quicker and easier to complete.
- The review team should be kept to a small, dedicated number of staff, to allow for consistency and familiarity of the tool and processes to develop over time. This proved to be beneficial from a resources and time investment vs. output point of view.
- The importance of a contextually relevant and appropriate AE/Harm classification system became increasingly apparent throughout the analysis. As comprehensive as the NCC MERP system is, the nuances of the EMS environment made application of this system difficult. The fit for purpose AE Severity Index, while more subjective, provided a simple and easy to implement alternative that warrants further examination.
- Documentation adequacy and legibility created difficulties in separating deficiencies in care from deficiencies in documentation. For such a system to be successful, it is essential that a review of documentation quality be carried out in parallel, or that a service have sufficient recourse to minimise the effect of poor documentation. The increased uptake of electronic medical records may limit the effects of some of these issues.
- Given the above-mentioned period of transition towards an electronic PCR, the topic of automation was discussed amongst the project team on several occasions. As the project progressed, it became increasingly apparent that the iterative manual review of cases over time added to the learning experience in trying to understand the interaction of triggers to adverse events and harm. It additionally offered insight into future potential trigger items, knowledge that would have otherwise been lost had the process become automated.

This study was amongst the first to test and analyse a trigger tool and its operational processes specific to the EMS setting. The incidence of trigger items observed in this analysis echoed the observations reported in the development of the EMS triggers, with the significant majority of trigger positive cases comprising the same three triggers. The incidence of AEs and Harm identified was relatively low, including the modified measures, when compared to the development of the EMS triggers. However, these differences could potentially be attributed to differences in sampling, with the focus on small consecutive samples in this analysis, compared to the single large sample focus with the initial development. The focus for this study was modelled on the sampling approach used by the GTT, which has shown reliable predictability compared with larger samples [11]. Nonetheless, the greater ease and shorter duration of time required to assess EMS PCRs, using the EMSTT, remains an avenue for further study employing larger samples.

No other peer-reviewed research exists examining the application of a TT over time, within the EMS setting. As such, there is no benchmark with which to compare the results observed in this study. Despite this, it stands to reason that variation in service models found in EMS around the world could have the potential to impact the results of the application of the EMSTT in other settings. This represents a significant opportunity for future research, to better understand the prevalence and incidence of AEs within EMS. While many in-hospital TTs exist, variation in patient cohorts, area of service, length of exposure to the healthcare service studied and varying outcome measures make comparison with the results of this study impossible.

## Conclusion

TTs represent a potential strategy towards the successful identification of the incidence of AE and harm within healthcare. With a better understanding of the case types and causes of AEs and Harm, targeted improvement projects can be designed and implemented to advance quality and patient safety. This study is amongst the first to test and analyse an EMS specific TT over time, in order to measure the rate of AE/Harm in this setting. With sufficient focus on implementation and data collection, and the inclusion of a contextually relevant classification system, the EMSTT represents a potentially successful strategy towards identifying the rate of AE/Harm within EMS.

## Additional files

Additional file 1: **Figure S1.** EMSTT Methodology Process. (TIFF 1205 kb)

Additional file 2: **Figure S2.** EMSTT Data Collection Process. (TIFF 1631 kb)

## Abbreviations

AE: Adverse events
EMS: Emergency medical services
EMSTT: Emergency Medical Services Trigger Tool
GTT: Global Trigger Tool
HMCAS: Hamad Medical Corporation Ambulance Service
IHI: Institute for Healthcare Improvement
NCC MERP: National Coordinating Council for Medication Error Reporting and Prevention
PCR: Patient Care Record
TT: Trigger Tool
