# Supplementary figures and images for: Application of the emergency medical services trigger tool to measure adverse events in prehospital emergency care: a time series analysis
Source: BMC Emerg Med. 2018 Nov 26;18:47. doi: 10.1186/s12873-018-0195-0 (PMC6258398; doi:10.1186/s12873-018-0195-0)

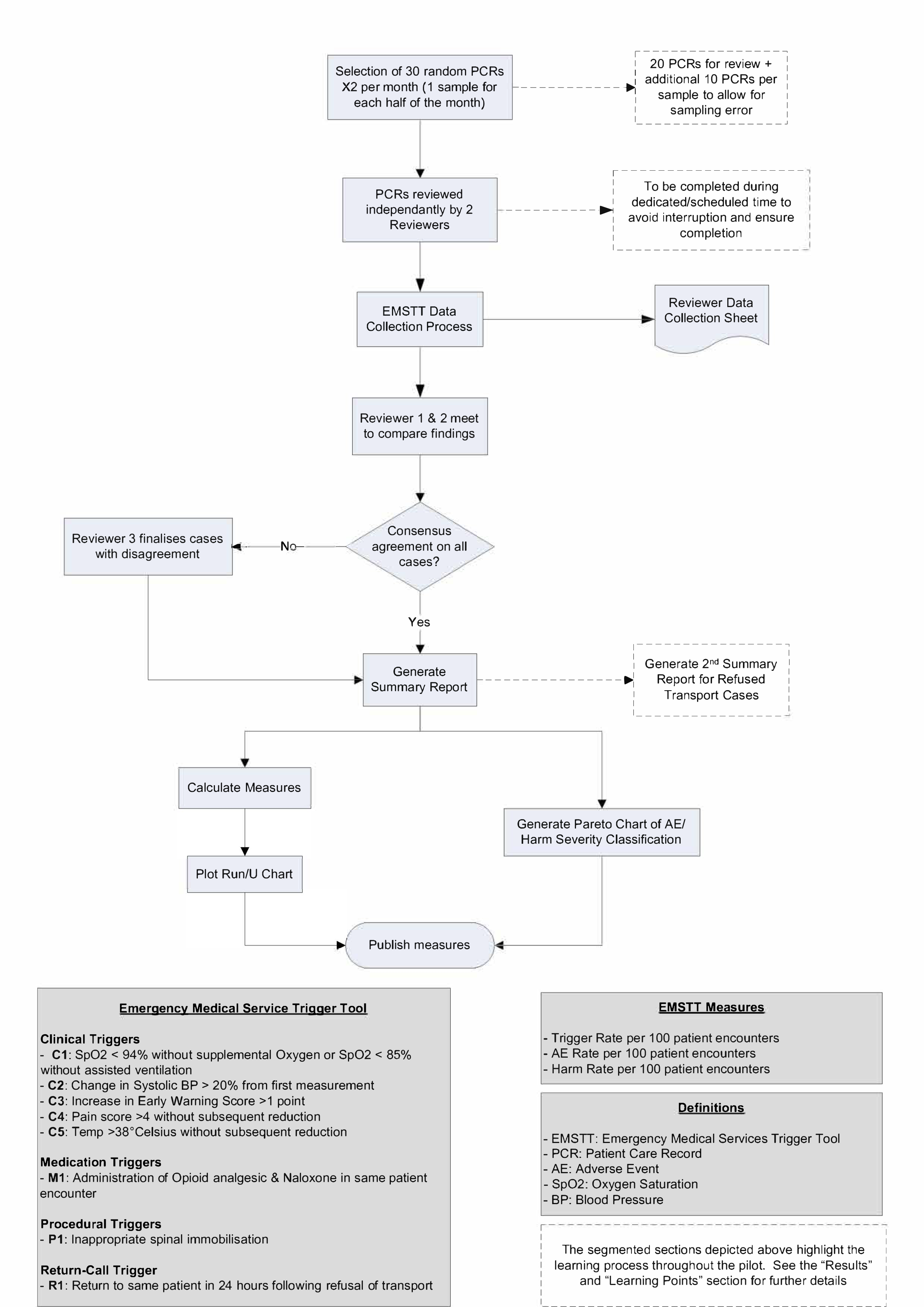

Supplement: Supplementary file 1 — Figure S1. EMSTT Methodology Process. (TIFF 1205 kb) [file 12873_2018_195_MOESM1_ESM.tiff]

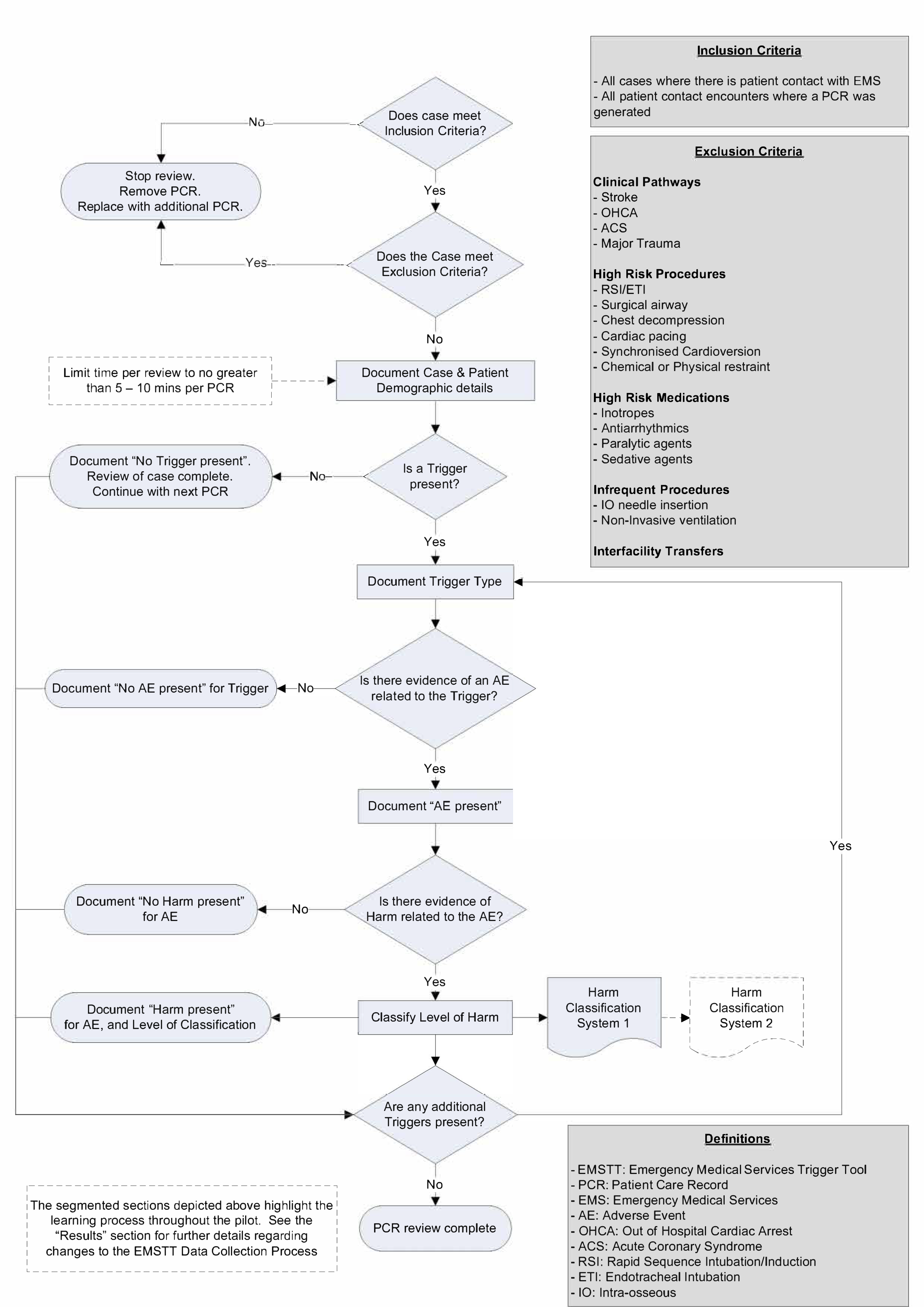

Supplement: Supplementary file 2 — Figure S2. EMSTT Data Collection Process. (TIFF 1631 kb) [file 12873_2018_195_MOESM2_ESM.tiff]
